# Ask Your Provider About Cannabis: Increasing Nurse Practitioner Knowledge and Confidence

**DOI:** 10.1089/can.2021.0061

**Published:** 2022-10-12

**Authors:** Tracy A. Klein, Ross Bindler

**Affiliations:** ^1^ College of Nursing, Washington State University Vancouver, Vancouver, Washington, USA.; ^2^ College of Nursing, Washington State University Spokane, Spokane, Washington, USA.

**Keywords:** CBD, medical therapy, pharmacology, THC

## Abstract

**Introduction:**

Nurse practitioners (NPs) are authorizing providers for medical cannabis in many states, and may serve as a primary care clinician. We report findings from a nationally distributed 2-h continuing education (CE) module aimed to improve knowledge, confidence, and willingness to communicate with patients about cannabis.

**Methods:**

Data were electronically obtained from the CE platform pre- and post-test (*n*=289) and a follow-up survey sent within 3 months postcompletion (*n*=184, 63%). Pre- and post-testing assessed cannabis pharmacodynamics, law, evidence-based use, metabolism, pharmacokinetics, laboratory testing, adverse reactions, and drug–drug interactions. The subsequent survey asked about changes in practice behavior, including willingness and self-identified recommendations for use. Quantitative and qualitative descriptive analysis and repeated-measures analysis of variance were used to analyze CE impact.

**Results:**

Significant improvement in scores was noted from pretest to post-test for all content with a mean improvement of 39.3% (95% CI: 30.6–47.9%). The greatest increases were for metabolism, pharmacokinetics, and drug–drug interaction content. At follow-up, 52.2% reported that the CE changed their attitudes about cannabis and although 86% had rarely or never applied it yet in practice, 92% reported they were now likely to inquire about cannabis use in their patients and 84% were likely to counsel patients about it. Although self-identified recommendations overlapped by conditions, some were unique to CBD (complex regional pain syndrome, migraine, mood disorder, smoking cessation) and THC products (appetite, cachexia, depression, fibromyalgia, HIV, seizure disorder, stress, and weight loss). Pain was the most common condition for recommendation of both CBD and THC, followed by anxiety and arthritis.

**Conclusions:**

NPs gained key knowledge about cannabis, which may impact patient care and prescribing practices. The educational module resulted in more willingness to discuss and counsel patients about cannabis, even if practitioner attitudes did not change.

## Introduction

An increasing number of states are legalizing cannabis for both medical and adult use.^1^ Obtaining medical cannabis for a specific diagnosed medical condition often begins with a written order from a licensed health care provider, such as a physician or nurse practitioner (NP), for the specific treatment of at least one approved health-based conditions in the state of residence. These approved conditions vary from state to state.^1^ Although some states use a dispensary system that includes a pharmacist, only 10 of the 36 states and four territories where cannabis is legal for medical use involve pharmacists in some aspect of counseling or dispensing of cannabis.^1–3^

Owing to the lack of a consistent pharmaceutical source, patients in locations of legalized cannabis will most frequently purchase cannabis from one or more community retailers with varying degrees of informational counseling. Although patients using cannabis for serious medical conditions such as cancer prefer to get information from their cancer team, <15% reported getting that information from a health care professional such as a doctor or nurse.^4^ Furthermore, there is documented reluctance of clinicians to authorize cannabis based on their own lack of knowledge, and many clinicians do not feel comfortable discussing cannabis in the clinical setting.^4–7^

When asked if they would like to learn more about cannabis to educate their patients, clinicians were often open to such learning.^6,7^ The need for high-quality clinical education regarding cannabis, particularly for medical use, has been recommended for all health professionals.^8,9^ Programs that train health professionals have not yet integrated curriculum regarding cannabis in their courses as a routine or expected competency.^10–12^ Conferences, medical literature, and websites are listed as common sources for information by clinicians who authorize cannabis.^11^ While admitting their own lack of knowledge and discomfort regarding cannabis and clinical use, health professionals still often seek other health professionals as their primary source of information.^6^ Educational gaps exist for medicine, nursing, and pharmacy students.^13^

## NPs and Cannabis Counseling

NPs are both primary and specialty care providers working in areas of practice, such as pain and mental health, where cannabis conversations occur. NPs are legally authorized to provide cannabis certification and authorization in many states. Prior studies on NPs and cannabis knowledge, practice, or attitudes include those completed in Canada^14^ and those that include NPs along with other health professionals.^13^ There are estimated to be >290,000 NPs in the United States.^15^

At present, 18 states authorize NPs to certify medical eligibility for cannabis.^16^ There are no reliable statistics available regarding how many NPs do authorize cannabis compared with those who may just recommend or discuss its benefits and risks and refer to other providers. There are additional limitations to seeking out education about, and initiating discussions of cannabis. In one study, 92% of NPs agreed that stigma is attached to recommending cannabis for medical use.^9^ Such stigma can further impact the likelihood of appropriate recommendation of cannabis for medical use.

In 2018 the National Council of State Boards of Nursing published a comprehensive document outlining guidelines for medical marijuana, *The NCSBN National Nursing Guidelines for Medical Marijuana*, which include recommendations for educational content for advanced practice registered nurses in their professional nursing programs.^17^ No study evaluates the subsequent integration of cannabis education into US NP educational programs since this recommendation. Lacking consistent curricular integration, NPs and other health care providers are likely to receive their structured education about cannabis from continuing education (CE) courses after they have already started in their practice.

## Purpose

We report the NP findings of a three-part national study focusing on improving communication between health care professionals and patients seeking information regarding use of cannabis for a medical condition. The overarching purpose of the “Ask your Provider About Cannabis” (APAC) project was to:

(1) Assess pre- and post-test knowledge of health care professionals regarding use of cannabis for medical symptoms
(2) Evaluate potential changes in practice reported after application of CE information
(3) Differentiate between health care professional suggested uses for cannabidiol (CBD) only versus cannabis products
(4) Increase willingness of health care professionals to communicate with patients regarding cannabis.

## Sample

A total of 841 participants registered for a national medical cannabis-focused CE program April 2020–March 2021 on an NP professional website with 839 providing a valid location (834 in the United States and four in Canada with one additional registrant serving in the armed services stationed in Europe). All participants who fully completed both the CE (*N*=289) and the pre- and post-test were sent a 3-month follow-up survey (Table 1). Of the total 289 who completed the pre- and post-test, 184 (63%) completed an additional follow-up survey assessing potential changes to practice. Sample participants were widely dispersed based on location of registered residency and represented participants from locations with medical and adult use cannabis, medical only, and restricted or no cannabis programs (Table 1).

**Table 1.** Continuing Education Registrants' State, or Territory, of Residence (*N*=839)

Authorized medical and adult use cannabis (*n*=205; 24.4%)	Medical cannabis only (*n*=365; 43.5%)	Restricted or no cannabis program (*n*=264; 31.5%)
Alaska (1)	Arkansas (12)	Alabama (12)
Arizona (31)^a^	Connecticut (12)	Georgia (28)
California (29)	Delaware (2)	Iowa (6)
Colorado (7)	Florida (53)	Idaho (1)
District of Columbia (1)	Hawaii (3)	Indiana (21)
Illinois (44)^a^	Louisiana (25)	Kansas (8)
Massachusetts (16)	Maryland (31)	Kentucky (20)
Maine (9)	Minnesota (14)	North Carolina (31)
Michigan (13)	Missouri (16)	Nebraska (9)
Northern Marianas (1)	Mississippi (30)^b^	South Carolina (11)
Montana (3)^a^	North Dakota (2)	Tennessee (26)
New Jersey (18)^c^	New Hampshire (8)	Texas (58)
Nevada (6)	New Mexico (12)	Virginia (21)
Oregon (5)	New York (33)^d^	Wisconsin (11)
South Dakota (4)^b^	Ohio (49)	Wyoming (1)
Vermont (3)	Oklahoma (9)	
Washington (14)	Pennsylvania (35)	
	Puerto Rico (1)	
	Rhode Island (6)	
	Utah (9)	
	West Virginia (3)	

Additional locations include Alberta (1), Armed Forces Europe (1), British Columbia (1), Ontario (2).

^a^ As of 2020.

^b^ Currently in court (2021) and not implemented.

^c^ As of 2021.

^d^ SB 854A legalizing adult use passed after data collection.

## Methods

A 2-h CE module “Medical and Recreational Cannabis: Research, Law, and Practice Considerations” was developed, mapped, and accredited using an NP/pharmacist educator team. Team expertise in cannabis and pharmacotherapeutic content included over 4 years of experience teaching graduate level pharmacology and pharmacokinetics, including cannabis, to pharmacists and NPs at two state universities. One expert also had extensive experience with a statewide evidence-based pharmaceutical information advice line, and two had published patient and health care provider-focused cannabis research.

Modules were further reviewed and approved by two state Boards of Pharmacy and by the accrediting bodies for both the American Association of Nurse Practitioners (AANP) and the Washington State Pharmacy Association (WSPA). Content focused on pharmacodynamics, cannabis law, evidence-based use, metabolism, pharmacokinetics, laboratory testing, adverse reactions, and drug–drug interactions. The recorded CE was hosted online by AANP and open without cost for CE credit. To measure simple learning objectives, a pre- and post-test was embedded and completed by all participants; answers from each question as well as total scores were compared through repeated-measures analysis of variance.

Within 3 months after completion of the CE, participants were sent a follow-up electronic survey and asked to assess changes to practice that could be attributed to the learning experience. Qualitative analysis using descriptive grouping further described and visualized participants' self-identified clinical recommendations for CBD and delta 9 tetrahydrocannabinol (THC) products in practice. Participants were offered the option of entering into a drawing for two 25.00 Amazon gift card upon completion of the follow-up survey and two were subsequently awarded. This study was deemed not human subjects research by the Washington University Institutional Review Board.

## Results

### Knowledge

New knowledge regarding cannabis and its use was achieved by CE completers, with 82.6% reporting that they knew less than half of the content before completion. The majority agreed that it strongly enhanced their current knowledge base (96.9%). There was a significant increase in scores in target pharmacologic content pre- and post-test in all areas (*p*<0.001, and a mean improvement of 39.3% (95% CI: 30.6–47.9%) (Table 2) with a marked increase in accuracy of response to questions about pharmacokinetics, metabolism, and drug–drug interactions.

**Table 2.** Pre- and Post-Test Assessment (*n*=289)

Questions	Correct responses [*n* (%)]		% Improvement	*p*-value
	Pretest	Post-test		
Q1: Pharmacodynamics	135 (46.7)	227 (78.5)	31.8	*p*<0.001^a^
Q2: Cannabis law	173 (59.9)	284 (98.3)	38.4	
Q3: Evidence-based use	61 (21.1)	182 (63)	41.9	
Q4: Metabolism	121 (41.9)	275 (95.2)	53.3	
Q5: Pharmacokinetics	97 (33.6)	233 (80.6)	47	
Q6: Laboratory testing	215 (74.4)	284 (98.3)	23.9	
Q7: Adverse reactions	186 (64.4)	269 (93.1)	28.7	
Q8: Drug–drug interaction	133 (46)	275 (95.2)	49.2	
	Mean pretest score (SD)=48.5% (17.1). Mean posttest score (SD)=87.8% (12.4).			

^a^ Significant improvement in scores noted from pretest to post-test with a mean improvement of 39.3% (95% CI: 30.6–47.9%).

### Changes in practice

Both the post-test (immediate) and follow-up survey (within 3 months of completion) assessed changes in attitudes and changes in practice since completing the CE module. Directly after completing the CE (post-test), most identified that knowledge gained either reinforced their practice or had potential to change it (69.9%), with 10.7% wanting additional information and 19.4% anticipating no changes to current practice (Table 3). When asked to reflect if the CE had changed their attitude about cannabis since application to practice, 52.2% reported it had, whereas 17.4% were unsure and 29.9% reported it had not. Most participants had not directly applied the CE content to their practice (37.5%) or had applied it less than five times (48.4%) (Table 3).

**Table 3.** Changing Attitudes and Practice Around Cannabis

Question	Response options	*n* (%)
Did the program change your attitudes on cannabis?^a^	No	55 (29.9)
	Unsure	32 (17.4)
	Yes	96 (52.2)
How often have you used the information from the CME in practice?^a^	Never	69 (37.5)
	1–5 times	89 (48.4)
	6–10 times	10 (5.4)
	11–15 times	7 (3.8
	16–20 times	3 (1.6)
	20 or more times	3 (1.6)
How likely will you inquire about cannabis use in patients?^a^	Highly unlikely	3 (1.6)
	Unlikely	7 (3.8)
	Likely	84 (45.7)
	Highly likely	86 (46.7)
How likely are you to counsel patients about cannabis?^a^	Highly unlikely	2 (1.1)
	Unlikely	28 (15.2)
	Likely	93 (50.5)
	Highly likely	57 (31)
After participating in this activity, do you intend to change your practice behavior?^b^	No changes	56 (19.4)
	Need more information	31 (10.7)
	Make Changes	115 (39.8)
	Practice reinforced	87 (30.1)

^a^ Assessed on 3-month follow-up survey (*n*=184).

^b^ Assessed directly following completion of CME (*n*=289).

### Self-identified recommendations

The follow-up survey offered both free text and structured choice options. In free text questions, participants were instructed to: “Please identify any diseases or ailments for which you have recommended CBD only in the past month” (*n*=122 responses) and “Please identify any diseases or ailments for which you have recommended cannabis in the past month” (*n*=122 responses). Although there was overlap in self-identified recommendations by conditions, there were some conditions unique to recommendations for CBD-only products (complex regional pain syndrome, migraine, mood disorder, smoking cessation) and THC products (appetite, cachexia, depression, fibromyalgia, HIV, seizure disorder, stress, and weight loss). Pain was the most common condition for recommendation of both CBD and THC, followed by anxiety and arthritis (Table 4).

**Table 4.** Reported Conditions for Patient Recommendation (*n*=122)

Q9 Cannabis	Q8 CBD Only	Q8 and Q9 (Total *N*)
Anorexia or appetite (4)	Anorexia or appetite (1)	5
Anxiety (10)	Anxiety (7)	17
Arthritis (6)	Arthritis (7)	13
Cancer and cancer-related symptoms excluding pain (3)	IBD (1)	4
Crohns or IBD (3)	Insomnia or sleep (3)	6
Depression (2)	Migraine (3)	5
Fibromyalgia (1)	Mood disorder (1)	2
Headaches (1)	Nausea (2)	3
HIV (1)		1
Insomnia or sleep (8)		8
Muscle spasms (1)		1
Nausea (1)		1
Pain	Pain	43
• Cancer related (3)	• Cancer related (2)	
• Chronic (9)	• Chronic (7)	
• Musculoskeletal (2)	• Complex regional pain syndrome (1)	
• Neuropathy (1)	• Joint (2)	
• Unspecified (7)	• Musculoskeletal (2)	
	• Unspecified (7)	
Post-traumatic stress disorder (4)	Post-traumatic stress disorder (4)	8
Seizure disorder (1)	Smoking cessation (1)	2
Stress (unspecified) (1)		1
Weight loss or noncancer cachexia (2)		2

CBD, cannabidiol; IBD, inflammatory or irritable bowel disorder.

### Willingness

Regardless of whether or not respondents had changed their own attitudes or actually recommended cannabis, 92.4% reported now being likely or highly likely to inquire about cannabis use in patients and 81.5% reported being likely or highly likely to counsel patients about cannabis use.

## Discussion

Health care professionals are interested in learning more about cannabis, whether or not they plan to advise its use. In this study, NPs reported that they are more likely to inquire about cannabis use and counsel patients regarding it after completing an educational program, although many had not had the opportunity yet to do so. Furthermore, the educational module resulted in more willingness to discuss and counsel patients about cannabis, even if practitioner attitudes did not change. Pre-test knowledge of cannabis overall was also low for NPs (mean pre-test score of 48.5% correct) despite many being located in states with legal medical, adult use, or both.

Knowledge of cannabis drug–drug interactions, pharmacokinetics, and metabolism content areas that had the largest knowledge increase after completion of this educational program. Because NPs have prescriptive authority in every state, knowledge and openness to discussing how cannabis that patients are using can impact medications, regardless of whether it is classified as “medical” or self-initiated, is critical for informed discussion and prescribing decisions.

Although knowledge about cannabis improved in all targeted content areas, one area identified for improvement is knowledge of the evidence supporting use for specific medical conditions. In the pre-test NPs scored the lowest (21.1%) in correctly identifying examples of evidence versus nonevidence-based cannabis medical recommendations. Based on the results from the post-CE survey after practice application, NPs were still recommending cannabis for some conditions that are not robustly supported by evidence such as the recommendation for cannabinoids for mental health disorders.^18^ However, many states offer discretion to the provider to decide whether and how a condition qualifies.^19^

Because state law varies considerably regarding approved conditions for authorization of cannabis, NPs who practice in more than one state may also experience confusion about overarching laws. Migraines, as an example, were most frequently mentioned for recommendation for CBD only (Table 4). This may reflect the status of the state law (migraines are an approved medical condition for cannabis in three states) or the restriction for this symptom in state law (migraines are an approved medical condition for low THC or no THC products in two states but not allowed for THC product certification).^19^ This may also reflect the constraints on both establishing and defining evidence around cannabis and its medical use with actual patients, owing to the current status of US law that impacts robust clinical trials and their funding.

### Limitations

There are limitations to studies that evaluate the link between CE and practice, particularly in both long-term change and measurement of patient-focused outcomes.^20^ The study design measures a brief time-limited reflection on practice, which may change with context and practice maturation. This study is subject to selection bias, as participants who completed both the CE and follow-up may differ particularly regarding their attitudes about cannabis from those who did not. Although national in nature, the complexity and variance of state law limits generalizability of these findings. The respondent location collected may not reflect all states of practice as participants can have multiple practice settings and licenses. Respondents were asked about their self-identified recommendations for use of cannabis without further prompts to differentiate between whether and how such recommendations aligned with evidence or current state law. This limits interpretation regarding whether additional constraints or facilitators influenced their clinical recommendations.

## Conclusions

NPs in the United States are likely to encounter patients who are either using cannabis, curious about cannabis, or inquiring about a friend or relative's use. Academic programs as of this writing do not uniformly prepare health care providers such as NPs for practice with cannabis. Studies from Canada recommend that nursing regulatory bodies should develop educational competencies specific to cannabis for therapeutic purposes.^14^ The National Council of State Boards of Nursing published its comprehensive guidance specific to medical cannabis in 2018, for both advanced practice and registered nursing.^17^ Despite this resource, integration into academic programs is inconsistent, and challenging within a state-based regulatory structure. Even with a national system of legalization as in Canada, NP scope of practice can be regulated at provincial level, and subsequent evaluation of Canadian cannabis legalization shows broad variance from province to province in both policy and academic integration.^21^

This national study contributes to understanding areas that may be particularly critical to target regarding education of health care providers regarding cannabis use for medical symptoms. In particular, we demonstrate the benefit of receiving CE modules in both improved knowledge scores and greater likelihood of talking with patients, provide information on where to target education such as on how evidence may be incorporated into discussions about cannabis, and show that further research is needed to identify why or what barriers are preventing practitioners to implementing this education into their clinical practice.

Normalization of discussion about cannabis can and should take place in a medical setting, regardless of whether cannabis is self-directed. Participants who completed this CE showed greater willingness to discuss cannabis with patients, even if they had not had the opportunity to apply it yet in the practice setting or had not changed their own attitudes. Increasing provider confidence and consistency in communication about cannabis, particularly in states where the law is ambiguous or changing, may be assisted by inclusion of complementary methods of learning that do not require active patient encounters. These include case studies, simulation, or standardized patients, which incorporate both symptom assessment and information regarding evaluating cannabis-focused evidence or practice-based guidelines. Further exploration is suggested regarding why and how attitude changes regarding cannabis occur as health care professionals accommodate to contextual changes such as changes in state law or practice setting, to confidently evaluate their patient's use of cannabis for medical symptoms.

## Abbreviations Used

AANP: American Association of Nurse Practitioners
CBD: cannabidiol
CE: continuing education
NP: nurse practitione
THC: tetrahydrocannabinol
WSPA: Washington State Pharmacy Association
